# Dose-Dependent Effects of Short-Chain Fatty Acids on 3T3-L1 Adipocyte Adipokine Secretion and Metabolic Function

**DOI:** 10.3390/nu17030571

**Published:** 2025-02-04

**Authors:** Ala Alzubi, Hannah X. Glowacki, Jessie L. Burns, Kelsey Van, Jamie L. A. Martin, Jennifer M. Monk

**Affiliations:** Department of Human Health and Nutritional Sciences, University of Guelph, Guelph, ON N1G 2W1, Canada

**Keywords:** short-chain fatty acids, acetate, propionate, butyrate, adipocytes, inflammation, adipokines, lipopolysaccharide, insulin-stimulated glucose uptake

## Abstract

Background: Short-chain fatty acids (SCFAs) produced from microbial fermentation of non-digestible carbohydrates and protein have been shown to modulate adipocyte adipokine secretion and metabolic function, which has implications for mitigating dysfunction in obese adipose tissue; however, the individual effects of different SCFAs and the optimal concentration required is unknown. The purpose of this study was to dose-dependently determine the effects of individual SCFAs on adipocyte adipokine secretion and metabolic function. Methods: We recapitulated the obese adipocyte inflammatory conditions using mature 3T3-L1 adipocytes and a physiological concentration of lipopolysaccharide (LPS) ± individual SCFAs, namely acetate, propionate, and butyrate, in a dose-dependent manner (0.25 mM, 0.5 mM, and 1 mM) for 24 h. Results: SCFAs dose-dependently affected inflammatory adipokine secretion, wherein at 1 mM, all three SCFAs reduced the secretion of leptin, IL-6 and IL-1β, but only propionate and butyrate reduced MCP-1/CCL2 and MIP-1α/CCL3 compared to control (*p* < 0.05). Interestingly, 1 mM acetate increased RANTES/CCL5 secretion versus control, whereas propionate and butyrate decreased RANTES/CCL5 secretion, and only 1 mM propionate reduced MCP-3/CCL7 secretion (*p* < 0.05). At the lower 0.5 mM concentration, both propionate and butyrate reduced IL-6 and IL-1β secretion compared to control (*p* < 0.05), and there was no difference in adipokine secretion between groups at the 0.25 mM SCFA concentration (*p* > 0.05). Intracellular protein expression in the ratio of phosphorylated–to–total STAT3 was reduced by all SCFAs at 1 mM and by propionate and butyrate at 0.5 mM versus control (*p* < 0.05). The ratio fo phosphorylated–to–total NFκB p65 expression was reduced by propionate and butyrate at 1 mM and by butyrate alone at 0.5 mM compared to control (*p* < 0.05). Basal (no insulin stimulation) and insulin-stimulated glucose uptake did not differ between control and any 1 mM SCFA treatment conditions (*p* > 0.05). Conclusions: Individual SCFAs exert different dose-dependent effects on LPS-stimulated adipocyte function.

## 1. Introduction

The local (i.e., within adipose tissue) and systemic obese phenotype is associated with chronic low-grade inflammation which perpetuates metabolic dysfunction including insulin resistance and dyslipidemia [1,2,3,4,5]. Dietary interventions that can attenuate the severity of the obese inflammatory phenotype provide an alternative approach for obesity management [6,7]. These approaches include the role of dietary non-digestible carbohydrates (NDCs) that are fermented by the gastrointestinal microbiome to produce short-chain fatty acids (SCFAs); predominantly acetate (or acetic acid), propionate (or propionic acid), and butyrate (butyric acid), although SCFAs with longer carbon chains such as valerate (or valeric acid) or caproate (or caproic acid) are also produced in lower amounts [8,9]. Additionally, SCFAs can be produced in lower amounts from the fermentation of undigested protein [10]. SCFAs have been shown to exert extraintestinal effects, although circulating concentrations are lower than fecal or gastrointestinal luminal concentrations [11,12,13,14,15,16].

With respect to modulating the severity of the obese phenotype, NDC supplementation in both humans and obese rodents has been shown to improve critical elements of the obese phenotype, including reducing weight gain [17,18,19,20,21,22,23,24,25,26,27,28,29], reducing adipose tissue mass [18,19,28,29,30,31,32], and improving blood glucose regulation and/or insulin resistance [26,33,34,35,36,37,38,39]. Production of SCFAs from dietary NDC intake likely underlies the beneficial effects, and SCFAs have been shown to modulate host metabolic function extra-intestinally in adipose tissue, skeletal muscle, liver, and the pancreas [10,40,41,42,43,44,45,46,47]. Specifically, within adipose tissue or adipocyte cultures, SCFAs have been shown to decrease lipolysis and increase triglyceride accumulation, along with increasing glucose uptake and adipogenesis [48,49,50,51,52,53,54]. Investigations into the metabolic effects of SCFA in adipose have primarily focused on select individual SCFAs, such as only acetate [49,50,53], only butyrate [48], or studies assessing differing outcomes between acetate, propionate, and butyrate [47,55,56,57], or SCFAs with a longer carbon chain length, such as valerate [54]. Although frequently considered to exert similar biological effects, in part due to their shared signaling through G protein-coupled receptors (GPCR, mainly GPR41, GPR43, and GPR109a), individual SCFAs have different binding affinities for these receptors [58]. Moreover, SCFAs are produced through microbial fermentation at different amounts (typically a 3:1:1 molar ratio of acetate–propionate–butyrate, respectively) that collectively account for 90–95% of colonic SCFA [9,59,60,61]. Furthermore, the amount and types of SCFA produced will vary based on multiple factors including the amount and type(s) of NDC in the diet (i.e., SCFA precursors including, but not limited to, soluble fibers, resistant starches, oligosaccharides, etc.), the relative fermentability of each type of NDC by the microbiota (i.e., fast versus slow fermentation), the microbiota composition of SCFA-producing microbial species, and intestinal SCFA absorptive capacity [10,62,63,64,65,66,67]. Previous comparative studies have reported that individual SCFAs exert differential effects on metabolic function and/or inflammatory mediator secretion in skeletal muscle cells [68,69] and adipocytes or adipose tissue [70,71].

The effect of SCFA on adipokine (i.e., adipose tissue-derived hormones, cytokines, and chemokines) gene expression or protein secretion from adipose tissue or adipocyte cultures has been focused on leptin and adiponectin, with some studies demonstrating the ability of select SCFAs to stimulate leptin gene expression or secretion from obese adipose tissue, with the effects of acetate differing from SCFAs with a longer carbon chain length [71,72], or having no effect of SCFA on leptin secretion [12]. In contrast, select SCFAs had no effect on adiponectin gene expression in obese primary adipose tissue; however, adiponectin gene expression increased with SCFA treatment or adipose tissue from obese donors with concurrent type 2 diabetes, thereby indicating that the adipose tissue microenvironment and metabolic status may impact how the tissue responds to SCFA signaling [72]. Conversely, acetate provided at higher doses (10 mM) has been shown to stimulate adiponectin secretion from primary brown adipose tissue and white adipose tissue [12], which indicates that the specific effects of SCFA may be dose dependent. The variations in SCFA dose and experimental conditions may account for differing outcomes. Moreover, an array of other cytokines and chemokines that can influence adipose tissue function remain under investigation, particularly at concentrations that mimic circulating SCFA levels in humans. What has been shown is the capacity for a 3 mM concentration of propionate to reduce inflammatory cytokine (TNFα) and chemokine (regulated upon activation, normal T cell expression, and secreted (RANTES)/chemokine ligand (CCL)5, macrophage inflammatory protein (MIP)-1α/CCL3 and MIP-1β/CCL4) secretion from obese primary adipose tissue cultures [73]. Separately, a lower and more physiologically relevant concentration (1 mM) of butyrate was shown to reduce adipocyte secretion of multiple inflammatory mediators including IL-6 IL-1β, MIP-1α/CCL3, RANTES/CCL5, macrophage chemoattractant protein (MCP)-1/CCL2, and MCP-3/CCL7 in response to combined normoxic and lipopolysaccharide stimulated conditions, whereas valerate only reduced IL-6 secretion under the same environmental conditions [70]. Furthermore, under hypoxic conditions, butyrate prevented and valerate increased adipocyte secretion of inflammatory mediators [70], thereby highlighting the different effects of individual SCFAs and the differing effects of SCFAs within unique obesity-associated adipose tissue microenvironments [72].

Since the range of SCFA concentrations in the systemic circulation is considerably lower than fecal levels [11,12,13,14,15,16,59,60,61], and the range of individual SCFAs evaluated at various concentrations differs between studies [57,72,74,75,76,77,78,79,80,81,82], and frequently, but not always, exceeds circulating concentrations, the purpose of this study was to assess the effects of the main SCFAs (acetate, propionate, and butyrate) [9,59,60,61] on adipocyte adipokine secretion and metabolic function in a dose-dependent manner.

## 2. Materials and Methods

### 2.1. T3-L1 Cell Culture and Differentiation

3T3-L1 murine pre-adipocytes (CL-173; American Type Culture Collection, Manassas, VA, USA) were cultured and maintained according to the manufacturer’s instructions in Dulbecco’s modified Eagle’s medium (DMEM; HyClone, Logan, UT, USA) supplemented with 4 mM L-glutamine, 4500 mg/L glucose, 10% (*v*/*v*) low endotoxin sterile-filtered fetal bovine serum (FBS; Millipore-Sigma, Oakville, ON, Canada) and 1% (*v*/*v*) penicillin-streptomycin (Fisher Scientific, Mississauga, ON, Canada), as described previously [70,83]. Pre-adipocytes were differentiated into adipocytes using DMEM containing 1 µmol/L dexamethasone, 0.5 mM 3-isobutyl-1-methylxanthine, and 10 µg/mL insulin (all from Millipore-Sigma), and matured in DMEM supplemented with 10 µg/mL insulin, as described previously [70,83]. Media were changed every two days. On day 8 post-differentiation, adipocytes were incubated for 12 h in serum-free DMEM containing 1% (*v*/*v*) penicillin-streptomycin prior to the addition of the experimental treatments.

### 2.2. Experimental Treatment Conditions

Adipocytes were incubated for 12 h in serum-free DMEM containing % (*v*/*v*) penicillin-streptomycin before they were used in any experiment. Subsequently, the cells were treated for 24 h with DMEM media alone (control, CON) or with media dose-dependently (0.25 mM, 0.5 mM, or 1 mM) containing each SCFA, namely sodium acetate (ACE), sodium propionate (PRO), and sodium butyrate (BUT) (all from Millipore-Sigma), concentrations that have been used previously [48,51,52,54,71,84]. These CON and SCFA-containing experimental treatment groups were either unstimulated (*n* = 6/experimental group) or treated with an inflammatory stimulus of 10 ng/mL lipopolysaccharide (LPS, from *Escherichia coli* 055:B5 (Millipore-Sigma); *n* = 9/experimental group). The dose of LPS utilized in these experiments recapitulates the circulating endotoxin levels reported in obese humans [85] and rodent high-fat-diet-induced obesity models [86,87]. Cell viability in response to all treatment conditions was assessed using Trypan blue exclusion and exceeded 90%, as seen previously [70]. After 24 h, culture supernatant was collected and stored at −80 °C to await secreted adipokine analysis, and cells were lysed using the lysis buffer from the RNA/Protein Purification Plus Kit (Norgen Biotek Corp., Thorold, ON, Canada), which was collected and stored at −80 °C.

### 2.3. Secreted Adipokines

Adipocyte secretion of adipokines in culture supernatant was measured by Bio-Plex using the Bio-Plex 200 system and accompanying Plex Manager software, version 6.0 (Bio-Rad, Mississauga, ON, Canada). All adipokines were measured following the manufacturers’ protocol, wherein adiponectin was measured separately using the Bio-Plex Pro Mouse Diabetes Adiponectin Assay (Bio-Rad, #171F7002M), and leptin and resistin were measured together using the Bio-plex Pro Reagent kit V (Bio-Rad, #12002798). Finally, secreted cytokines (IL-1β, IL-6, IL-10, and TNFα) and chemokines (MCP-1/CCL2, MCP-3/CCL7, MIP-1α/CCL3, MIP-1β/CCL4, and RANTES)/CCL5) were simultaneously measured using the mouse Bio-Plex Pro kit (Bio-Rad). IL-10 was below the assay limit of detection in all samples. Secreted protein levels for all other adipokines assessed in unstimulated 3T3-L1 cell cultures ± SCFA did not differ between experimental groups (i.e., CON, ACE, PRO, and BUT) and were significantly lower than LPS-stimulated secretion levels (*p* < 0.05; results not shown).

### 2.4. Intracellular Protein Analysis

Total intracellular protein was quantified using the bicinchoninic assay according to the manufacturer’s instructions (Thermo-Fisher Scientific, Mississauga, ON, Canada). An equal amount of protein (10 µg/sample/assay) was used to measure the ratio of phosphorylated-to-total expression of transcription factors STAT3 (phosphorylated STAT3 [Tyr705]–total STAT3) and NFκB p65 (phosphorylated NFκB p65 [Ser536]–total NFκB p65) using an enzyme-linked immunosorbent assay, as per the manufacturer’s instructions (Thermo-Fisher Scientific). The final absorbance was measured at 450 nm using SpectraMax M5e Multimode Plate Reader (Molecular Devices, San Jose, CA, USA).

### 2.5. Glucose Uptake Assay

Glucose uptake was measured in mature 3T3-L1 adipocyte cultures (±LPS and 1 mM ACE, PRO, or BUT) for 24 h (*n* = 6–8/treatment group) in both basal (i.e., non-insulin stimulated) and insulin-stimulated cultures (described below) using a colorimetric Glucose Uptake Assay Kit (Abcam, Waltham, MA, USA) according to the manufacturer’s instructions and as described previously [69]. Basal stimulated 3T3-L1 cell cultures were not treated with insulin but instead treated with phosphate-buffered saline for 20 min, followed by the addition of 10 mM of the glucose analog, 2-deoxy glucose, for 20 min at 37 °C. Insulin-stimulated 3T3-L1 adipocyte cultures were treated with 1 µM insulin (Millipore-Sigma) for 20 min, followed by the addition of 10 mM of 2-deoxy glucose for 20 min at 37 °C. At the end of the kit protocol, optical density was measured at 412 nm using a spectrophotometer (Molecular Devices, San Jose, CA, USA). Glucose-starved cell cultures (*n* = 4/treatment group) that did not receive either 2-deoxy glucose or insulin served as background negative control cultures, and there was no difference in optical density between the control and SCFA-negative control cultures.

### 2.6. Statistical Analysis

All data are expressed as means ± SEM. Data were analyzed by one-way ANOVA followed by Tukey’s multiple comparisons test for post hoc analysis between experimental treatment groups (*p* ≤ 0.05). The Shapiro–Wilk test was used to test for normality. All analyses were conducted using GraphPad Prism, version 10 (GraphPad Software, Inc., La Jolla, CA, USA).

## 3. Results

### 3.1. SCFAs Dose-Dependently Attenuate Inflammatory Adipokine Secretion in Response to LPS Stimulation

**Figure 1:**
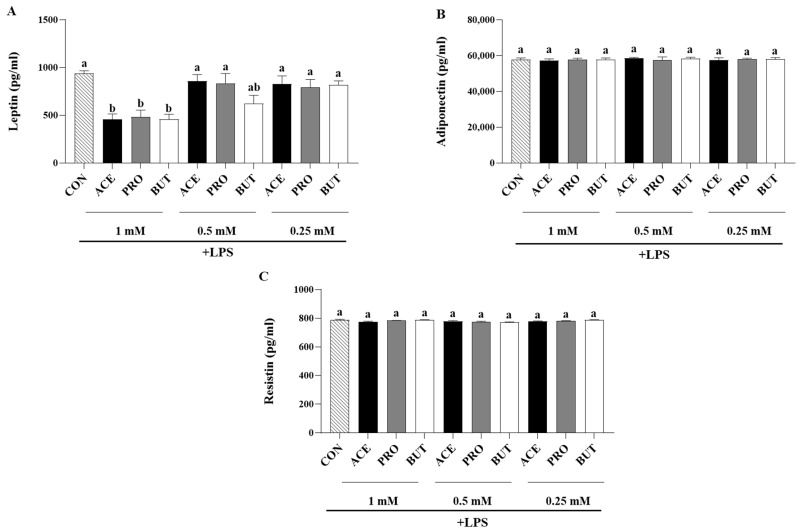
Individual SCFAs’ dose-dependent (1 mM, 0.5 mM, and 0.25 mM) effects on LPS-stimulated 3T3-L1 adipocyte secreted protein levels [(**A**): leptin, (**B**): adiponectin, and (**C**): resistin]. Bars represent mean values ± SEM (*n* = 9/experimental group). Data were analyzed via one-way ANOVA followed by Tukey’s multiple comparison test. Bars not sharing a lower-case letter differ (*p* < 0.05).

In response to LPS stimulation, all three SCFAs (ACE, PRO, and BUT) reduced leptin secretion compared to CON at the highest concentration tested (1 mM); however, at the lower SCFA concentrations assessed, there was no difference in leptin secretion between experimental groups (Figure 1A). There was no difference in the secretion of resistin (Figure 1B) or adiponectin (Figure 1C) between experimental groups at any SCFA concentration.

**Figure 2:**
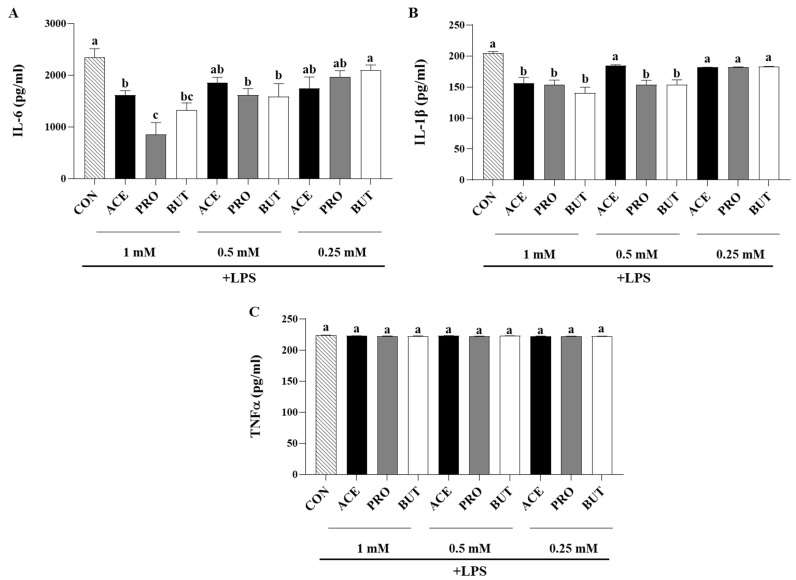
Individual SCFAs’ dose-dependent (1 mM, 0.5 mM and 0.25 mM) effects on LPS-stimulated 3T3-L1 adipocyte secreted protein levels [(**A**): IL-6, (**B**): IL-1β, and (**C**): TNFα]. Bars represent mean values ± SEM (*n* = 9/experimental group). Data were analyzed via one-way ANOVA followed by Tukey’s multiple comparison test. Bars not sharing a lower-case letter differ (*p* < 0.05).

Inflammatory cytokine secretion in response to LPS stimulation was differentially affected by each SCFA in a dose-dependent manner (Figure 2). ACE, PRO, and BUT all reduced IL-6 secretion at the highest concentration assessed (1 mM) compared to CON (*p* < 0.05; Figure 2A), wherein IL-6 secretion was the lowest from the PRO-treated adipocytes. At the middle SCFA dose (0.5 mM), only PRO and BUT reduced IL-6 secretion in comparison to CON (*p* < 0.05), and there was no difference in IL-6 secretion between any treatment groups at the 0.25 mM SCFA concentration (Figure 2A).

All three SCFAs reduced IL-1β secretion at the 1 mM dose compared to CON (*p* < 0.05; Figure 2B), while only PRO and BUT reduced IL-1β secretion at the middle dose (0.5 mM) compared to CON (*p* < 0.05), and there were no differences between treatment groups at the lowest dose (0.25 mM) (*p* > 0.05). There was no difference between treatment groups in the secretion of TNFα at any SCFA concentration assessed (*p* > 0.05; Figure 2C).

**Figure 3:**
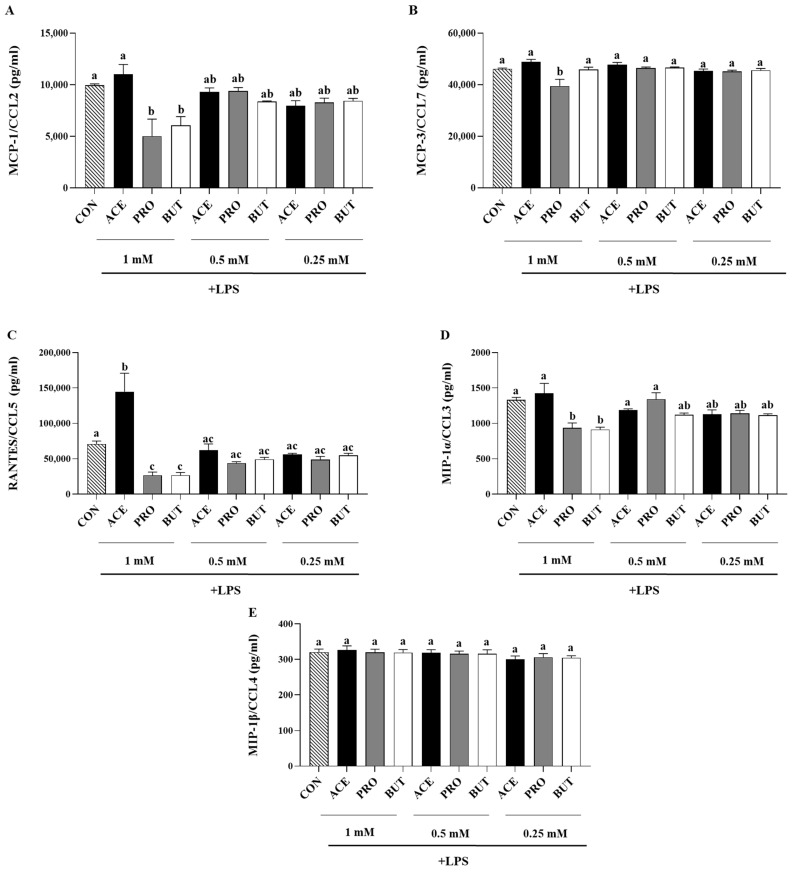
Individual SCFAs’ dose-dependent (1 mM, 0.5 mM, and 0.25 mM) effects on LPS-stimulated 3T3-L1 adipocyte secreted protein levels [(**A**): MCP-1/CCL2, (**B**): MCP-3/CCL7, (**C**): RANTES/CCL5, (**D**): MIP-1α/CCL3, (**E**): MIP-1β/CCL4]. Bars represent mean values ± SEM (*n* = 9/experimental group). Data were analyzed via one-way ANOVA followed by Tukey’s multiple comparison test. Bars not sharing a lower-case letter differ (*p* < 0.05).

Inflammatory chemokine secretion by adipocytes in response to LPS and SCFA is shown in Figure 3. MCP-1/CCL2 secretion was reduced by both PRO and BUT at the 1 mM concentration compared to CON (*p* < 0.05; Figure 3A), while ACE did not differ from CON (*p* > 0.05). There was no difference in MCP-1/CCL2 secretion between treatment groups at either the 0.5 mM or 0.25 mM SCFA treatment concentrations (*p* > 0.05). Only PRO reduced MCP-3/CCL7 secretion at the 1 mM concentration as compared to CON (*p* < 0.05; Figure 3B), and there were no differences between CON and PRO at the other lower concentrations assessed (*p* > 0.05). ACE and BUT did not differ from CON at any concentration tested compared to CON (*p* > 0.05). RANTES/CCL5 secretion was increased by ACE at the 1 mM concentration compared to CON (*p* < 0.05), while both PRO and BUT reduced RANTES/CCL7 secretion in comparison to CON (*p* < 0.05; Figure 3C).

MIP-1α/CCL3 secretion was reduced by both PRO and BUT at the 1 mM concentration compared to CON (*p* < 0.05; Figure 3D); however, there was no difference between CON and PRO or BUT at the lower concentrations, and ACE had no effect on LPS-stimulated secretion of MIP-1α/CCL3 at any concentration tested (*p* > 0.05). MIP-1β/CCL4 secretion did not differ between any treatment groups (*p* > 0.05; Figure 3E).

### 3.2. Transcription Factor Activation (Ratio of Phosphorylated-to-Total) Is Dose-Dependently Reduced by SCFA in Response to LPS Stimulation

**Figure 4:**
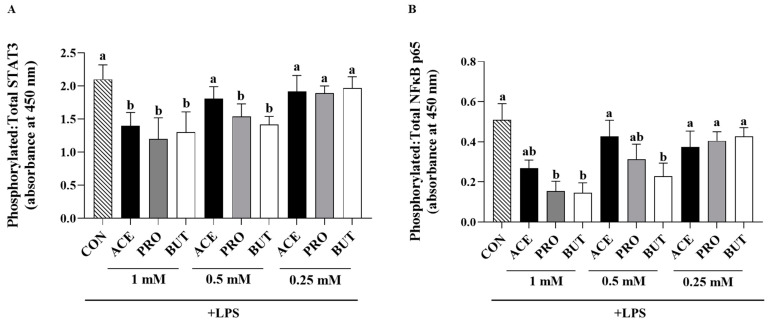
Individual SCFAs’ dose-dependent (1 mM, 0.5 mM, and 0.25 mM) effects on LPS-stimulated 3T3-L1 adipocyte intracellular protein levels ((**A**): phosphorylated–to–total STAT3, and (**B**): phosphorylated–to–total NFκB p65). Bars represent mean values ± SEM (*n* = 9/experimental group). Data were analyzed by one-way ANOVA followed by Tukey’s multiple comparison test. Bars not sharing a lower-case letter differ (*p* < 0.05).

Adipocyte intracellular protein expression of transcription factors, expressed as the ratio of phosphorylated (i.e., activated)-to-total, in response to LPS stimulation, is shown in Figure 4. All three individual SCFAs reduced intracellular protein expression of STAT3 at the 1 mM concentration compared to CON (*p* < 0.05; Figure 4A). At the 0.5 mM concentration, only PRO and BUT reduced STAT3 expression compared to CON (*p* < 0.05), whereas ACE had no effect at the 0.5 mM concentration. At the lowest concentration (0.25 mM) assessed, there was no difference in STAT3 expression between treatment groups (*p* > 0.05).

In comparison to CON LPS-stimulated adipocyte cultures, NFκB p65 intracellular protein expression was reduced by both PRO and BUT at the 1 mM dose (*p* < 0.05; Figure 4B); however, ACE did not differ from CON (*p* > 0.05). At the 0.5 mM dose, only BUT reduced intracellular expression of NFκB p65 compared to both CON and the other SCFAs (*p* < 0.05). There was no difference between CON and any SCFA at the 0.25 mM dose (*p* < 0.05).

### 3.3. Insulin-Stimulated Glucose Uptake in LPS-Stimulated Adipocytes Is Not Affected by SCFA

**Figure 5:**
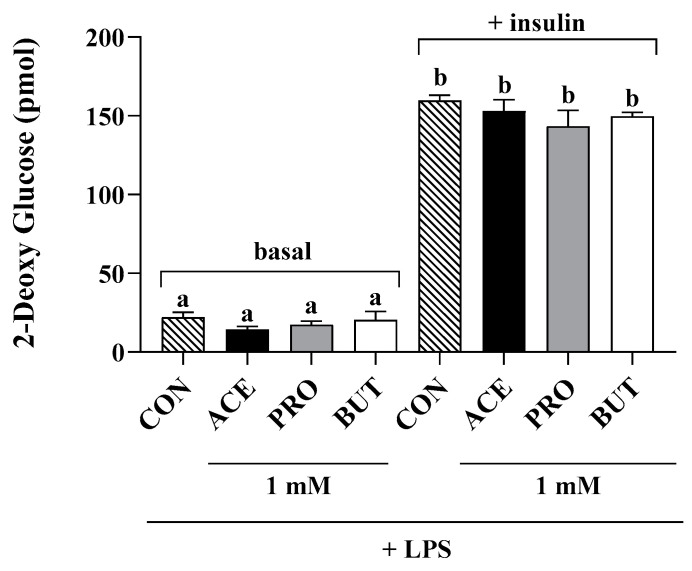
Effect of individual SCFAs (1 mM) on LPS-stimulated 3T3-L1 adipocyte glucose uptake under basal (i.e., non-insulin stimulated, phosphate-buffered saline for 20 min followed by 10 mM 2-deoxy glucose for 20 min at 37 °C) and insulin-stimulated conditions (1 µM insulin for 20 min followed by 10 mM 2-deoxy glucose for 20 min at 37 °C). Bars represent mean values ± SEM (*n* = 6–8/experimental group under basal and insulin-stimulated conditions). Data were analyzed by one-way ANOVA followed by Tukey’s multiple comparison test. Bars not sharing a lower-case letter differ (*p* < 0.05).

Glucose uptake in basal (i.e., non-insulin-stimulated) and insulin-stimulated adipocyte cultures was assessed in CON and only 1 mM SCFA-treated cultures in the presence of LPS, as shown in Figure 5. There was no difference in glucose uptake between any treatment groups (CON and individual SCFAs) in both the basal (i.e., non-insulin simulated) and insulin-stimulated conditions (*p* > 0.05).

## 4. Discussion

The results from this study demonstrate the dose-dependent effects of individual SCFAs on LPS-stimulated adipocyte adipokine secretion, activation status (i.e., ratio of phosphorylated–to–total) of transcription factors NFκB p65 and STAT3 that affect inflammatory mediator production, and glucose uptake (under both basal and insulin-stimulated conditions). The experimental approach utilized aimed to create an adipocyte cell culture microenvironment that more accurately recapitulated the conditions that obese adipocytes would experience in vivo by utilizing an LPS dose that mimics circulating LPS levels observed in obese humans [85] and rodents [86,87]. Furthermore, the doses of individual SCFAs used in this study (0.25–1 mM) more accurately reflect circulating SCFA levels in humans, which can be variable depending upon the underlying physiological condition [11,12,13,14,15,16,88,89]. Investigations into the metabolic effects of SCFA in adipose have primarily focused on select individual SCFAs, such as only acetate [49,50,53], only butyrate [48], or studies assessing differing outcomes between acetate, propionate, and butyrate [47,55,56,57], or consider the effects of SCFAs with a longer carbon chain length, such as valerate [54,70]. Importantly, the concentration of SCFA used in this study is within the range of SCFA concentrations observed in human blood [88,89], which highlights the importance of assessing SCFA-mediated effects at lower concentrations. Importantly, the 1 mM SCFA concentration used in this study has been shown previously to not affect adipocyte viability [54,70,71], which we also confirmed herein. In contrast, other studies assessing the metabolic effects of SCFA used higher concentrations that do not necessarily reflect circulating levels in vivo that adipose tissue would be more likely to encounter [49,56,57,74,84,90].

The cellular composition of obese adipose tissue within the stromal vascular compartment changes based on the infiltration of additional immune cells and their subsequent change in polarization and functional status [91,92], which is directly connected to tissue inflammation and metabolic dysfunction. It is equally important to identify the intact tissue adipokine secretory profile in response to individual SCFAs, and to identify the cellular source(s) of each adipokine secreted in response to SCFA. The current study investigated the effects of individual SCFAs on the adipocyte response to LPS-mediated stimulation, as adipocytes have been identified as a critical immunomodulatory cell type in adipose tissue that is responsible for not only adipokine production (hormones, cytokines, and chemokines to recruit additional immune cells to the adipose tissue), but also the release of proinflammatory lipids, extracellular matrix proteins, and extracellular vesicles that contribute to local and systemic inflammation and metabolic function [93]. The results from the current study highlight the effect of individual SCFAs on adipocyte-mediated adipokine secretion.

The spectrum of secreted inflammatory adipokines assessed in the current study included hormones, cytokines, and chemokines, which have been shown to contribute to obese adipose tissue low-grade chronic inflammation, increased immune cell recruitment, and subsequent metabolic dysfunction [94,95], while concomitantly also assessing the effect of SCFA on the secretion of anti-inflammatory and/or insulin-sensitizing mediators such as IL-10 and adiponectin [94,96]. The majority of studies investigating the role of SCFA on adipocyte or adipose tissue adipokine gene expression and/or protein secretion utilize different model systems, such as primary tissue explants or adipocytes alone, utilize a range of SCFA concentrations, and tend to be more focused on the adipokines leptin and adiponectin [72,97,98]. A higher concentration of propionate (3 mM) has been shown to reduce the secretion of some inflammatory mediators (cytokines and chemokines, namely TNFα, RANTES/CCL5, MIP-1α/CCL3, and MIP-aβ/CCL4) from primary adipose tissue cultures [77], whereas a lower 1 mM concentration of butyrate was shown to reduce the secretion of a spectrum of inflammatory cytokines and chemokines from LPS-stimulated adipocytes under normoxic conditions (namely IL-6, IL-1β, MCP-1/CCL2, MCP-3/CCL7, MIP-1α/CCL3, and RANTES/CCL4) and prevent the rise in inflammatory mediator secretion from hypoxic adipocytes [70]; however, in both studies, the effects of other individual SCFAs were not evaluated. In the current study, the majority of the effects of SCFA on adipokine secretion were observed at the 1 mM concentration, indicating that lower SCFA concentrations (0.25 mM) are insufficient to induce a change in adipocyte adipokine secretion. PRO and BUT (at 1 mM) exerted similar effects by reducing adipocyte secretion of leptin, IL-6, IL-1β, MCP-1/CCL2, RANTES/CCL5, and MIP-1α/CCL3 (Figure 1, Figure 2 and Figure 3), wherein a lower concentration (0.5 mM) of PRO and BUT also reduced IL-6 and IL-1β secretion (Figure 2). The reduced secretion of cytokines and chemokines in response to butyrate confirmed previous findings that highlighted functional differences between butyrate and valerate [70], and the current study highlights the functional similarities between BUT and PRO, with both SCFAs exerting an anti-inflammatory and anti-chemotactic effect in LPS-stimulated adipocytes. Importantly, BUT and PRO did not differ in the secretion of MCP-3/CCL7 (Figure 2), which demonstrates that not all SCFAs exert similar effects. In this connection, ACE was shown to reduce only leptin, IL-6, and IL-1β secretion; however, ACE significantly increased RANTES/CCL5 secretion (Figure 1, Figure 2 and Figure 3). Higher circulating levels of RANTES are associated with impaired glucose tolerance [81]. Obese adipose tissue expression of RANTES is positively correlated with the expression of the macrophage marker CD68 [99], as well as increased local T cell accumulation [100]. This indicates that ACE may contribute to immune cell chemotaxis to inflamed adipose tissue, whereas PRO and BUT exhibited an anti-chemotactic effect that could reduce immune cell recruitment into obese adipose tissue. Upon macrophage and T cell infiltration and polarization into inflammatory phenotypes in obese adipose tissue, these cells contribute to inflammatory mediator production, increased reactive oxygen species production, endothelial dysfunction, and metabolic dysfunction, in particular, insulin resistance [93,101,102]. Moreover, leptin, which is elevated in obese adipose tissue [103], has been shown to play a key role in directing chemokine production [101]; however, all SCFAs assessed were shown to reduce leptin secretion (Figure 1). Conflicting findings have been reported in primary adipose tissue using higher SCFA concentrations that increased leptin gene expression and secretion [72,97], or had no effect [98]. Conversely, adiponectin functions as an insulin-sensitizing and anti-inflammatory adipokine [104], whose secretion decreases in obese adipose tissue [105,106]. In the current study, adiponectin secretion did not differ between SCFA treatment groups (Figure 1); however, a high dose of acetate (10 mM) has been shown to stimulate adiponectin secretion from primary brown adipose tissue and white adipose tissue [98], and adiponectin gene expression has been shown to increase in primary adipose tissue from obese type 2 diabetic donors but had no effect on non-diabetic adipose tissue [72], which highlights that SCFAs may exert different effects based on the inflammation and metabolic status of the tissue and warrants further investigation. The addition of individual SCFAs at 5% *w*/*w* to a high-fat diet was shown to increase adiponectin gene expression [55], and the same study also observed an increase in resistin gene expression compared to high-fat diet controls. Resistin levels are increased in obesity [107,108]; however, in the current study, there was no difference in resistin secretion between treatment groups (Figure 1). Collectively, the results demonstrate dose-dependent anti-inflammatory and anti-chemotactic effects of SCFAs on the adipocyte secretory profile, with the strongest effects attributable to propionate and butyrate.

Activation of NFκB occurs downstream of LPS signaling via Toll-Like Receptor (TLR)-2 and TLR4 in adipocytes and serves as a critical transcription factor controlling the gene expression of many inflammatory cytokines and chemokines (e.g., TNFα, IL-6, MCP-1/CCL2, etc.) that contribute to obesity-associated inflammation and metabolic dysfunction [109,110]. All SCFAs reduced NFκB p65 activation (ratio of phosphorylated-to-total cellular protein) at the 1 mM dose, wherein the secretion of key inflammatory adipokines that contribute to adipocyte metabolic dysfunction, such as IL-6 and MCP-1/CCL2 [94,95], was concomitantly also reduced (Figure 1, Figure 2, Figure 3 and Figure 4). Furthermore, only BUT reduced NFκB p65 activation at the 0.5 mM concentration, highlighting a dose-dependent effect of SCFAs on transcription factor activation. Similarly, STAT3 is another transcription factor that influences inflammatory adipokine gene expression and immune and metabolic functions in adipose tissue [111,112] and was dose-dependently reduced by each individual SCFA. At the 1 mM concentration, all three SCFAs reduced phosphorylated–to–total STAT3 intracellular protein levels, whereas both PRO and BUT reduced STAT3 activation at the 0.5 mM concentration, and there was no effect at the lowest concentration (0.25 mM) assessed (Figure 4). Collectively, these findings mirrored the adipokine secretion profile attributable to each SCFA, with butyrate and propionate having the strongest effects, particularly at lower concentrations compared to acetate.

Despite the connection between increased obese adipose tissue inflammatory adipokine production and metabolic dysfunction [94,95], and the anti-inflammatory and anti-chemotactic effects of predominantly PRO and BUT (and to a lesser degree ACE), insulin-stimulated glucose uptake did not differ between control and SCFA treatments in LPS-stimulated adipocytes (Figure 5). Supplementation of a high-fat diet with 5% *w*/*w* sodium butyrate was shown to reduce fasting insulin levels, the response to an insulin tolerance test, and insulin sensitivity assessed by HOMA-IR [113]. Previously, propionate (at concentrations ranging from 1 to 10 mM) was shown to increase insulin-stimulated glucose uptake, whereas the effects of butyrate to improve insulin sensitivity were only observed at a 10 mM concentration [114]. Moreover, in the same study, propionate and butyrate were shown to dose-dependently inhibit lipolysis and de novo lipogenesis, with the strongest effects observed at supraphysiological concentrations (10 mM) [114], indicating that SCFAs may also impact other metabolic functions beyond glucose uptake. In this connection, high concentrations of acetate (6.2 mM) and propionate (3.2 mM), but lower concentrations of butyrate (0.8 mM), that are closer to those used in the current study, have been shown to promote lipid accumulation in adipocytes [115]. Further studies are required to dose-dependently investigate the breadth of potential metabolic effects attributable to individual SCFAs, and to determine both the kinetics of those effects, and if those effects are a direct consequence of SCFA signaling (i.e., through GCPR) or through altering adipokine levels within the adipose tissue microenvironment. The translational potential of this work can be increased by future studies that utilize primary adipose tissue, which can more accurately reflect in vivo conditions and comprises diverse cell types that are present in adipose tissue, in contrast to the limited scope of the current study that utilized only adipocytes from an immortalized cell line.

In order to accurately provide recommendations for dietary intakes of SCFA precursors, it is necessary to identify the SCFA concentration that is required to exert beneficial effects within the extra-intestinal tissues, such as adipose. The ability of individual SCFAs to beneficially modulate adipocyte function via anti-inflammatory and anti-chemotactic mechanisms identifies the therapeutic potential of SCFAs to improve obese adipose tissue function. Investigating strategies to increase SCFA levels, either through nutritional interventions to increase NDC dietary intakes and/or directly providing SCFA via supplementation approaches hold potential for attenuating adipose tissue dysfunction associated with obesity, although further study is required. Using physiologically relevant cell culture models can identify the mechanisms and relevant concentrations that are required to beneficially affect adipocyte function, which can be further tested within intact adipose tissue in future studies in a directed manner. Given the variables that can affect SCFA production in vivo, including the amount, type, and fermentability of each NDC consumed, the relative abundance of SCFA-producing microbial species found within the microbiota, and the absorptive capacity of the intestinal epithelium [10,62,63,64,65,66,67], comprehensive assessments of adipocyte functions using physiologically relevant SCFA concentrations [11,12,13,14,15,16,88,89] can help to address this knowledge gap and can demonstrate the potential for individual SCFAs to beneficially affect adipocyte function.

## 5. Conclusions

Individual SCFAs exert differential effects on LPS-stimulated adipocyte function, wherein propionate and butyrate exert a strong dose-dependent anti-inflammatory and anti-chemotactic effect on transcription factor activation and the adipokine secretory profile compared to acetate.

## Data Availability

Data are contained within the article.
